# Rheumatic Pericarditis and Its Consequences

**Published:** 1907-11-09

**Authors:** J. Walter Carr

**Affiliations:** Physician to the Royal Free Hospital and to the Victoria Hospital for Children, Chelsea.


### RHEUMATIC PERICARDITIS AND ITS CONSEQUENCES.

By J. WALTER CARR, M.D., F.R.C.P., Physician to the Royal Free Hospital and to the Victoria Hospital for Children, Chelsea.

Very noteworthy is the change during recent years in the views of the medical profession in regard to rheumatic fever and its so-called complications. Some 20 or 30 years ago the disease was described almost entirely from the standpoint of its symptoms and course in adults, the result being that it was looked upon as essentially an acute inflammation of the synovial membrane of the joints, with endocarditis and pericarditis as more or less frequent complications. Increased knowledge of the disease as it manifests itself in childhood, together with modern views as to its infective nature, have largely revolutionised this idea, have taught us that acute rheumatism is far more than a disease merely of the joints, and that in early life particularly the heart rarely escapes damage, whilst the joints may be but slightly if at all affected; in fact, that from the clinical standpoint cardiac inflammation is by far the most frequent and important manifestation of rheumatic fever in children, and that the synovitis may well be regarded as simply a complication.

The rheumatic poison may attack any part of the heart: endocarditis, affecting especially the mitral valve, is the most frequent lesion, and certainly the one which attracts most attention, but it is doubtful whether the myocardium ever entirely escapes, though unfortunately we have no certain means of diagnosing its presence during life. Pericarditis is undoubtedly less common, but, for reasons to be presently mentioned, is perhaps the most serious lesion of all. It is apt to be overlooked, even when subacute or acute, and still more frequently when it has given rise to its after-consequence, adherent pericardium. One of the first points, therefore, to remember about rheumatic pericarditis is its insidious character. We are still to some extent in bondage to the old descriptions of acute pericarditis, with the account of intense pain, urgent dyspnœa, high fever, sleeplessness, delirium, and so forth. Cases of this kind are no doubt occasionally met with, but far more frequently the symptoms are by no means conspicuous—trifling præcordial discomfort, and sometimes not even that, only slight dyspnœa so long as the patient keeps fairly quiet, very little pyrexia, and no delirium. Not uncommonly no suspicion of pericarditis is entertained until a routine physical examination discloses the presence of friction; sometimes the disease develops while the patient is under treatment in hospital, and its existence is discovered almost accidentally. At times, doubtless, it passes altogether unnoticed, for in many cases in which adherent pericardium is diagnosed or suspected during life, and found to be present post-mortem, the most careful inquiry from the patient and his friends may have failed to elicit any facts whatever suggestive of a former attack of pericarditis. Cheadle has pointed out that " rheumatic pericarditis in early life is apt to be sub-acute, persistent, recurrent, and progressive, going on for weeks or months." Sometimes the area over which friction is audible is very limited; quite recently I have seen a case in which it was restricted to the immediate neighbourhood of the ensiform cartilage, and in this patient there was absolutely no complaint of pain. The disease ended fatally after a few weeks.

A large effusion is comparatively rare, consequently paracentesis pericardii is very seldom required. It is true that the area of cardiac dullness is sometimes greatly increased, but this is due more to dilatation of the heart itself than to effusion around it. Perhaps a more reliable test than the area of cardiac dullness for the presence of effusion in any considerable quantity is afforded by physical signs of consolidation at the base of the left lung. In some cases a helpful sign of effusion, described by Ewart, is a quadrilateral area of dullness adjacent to the spinous processes of the lowest dorsal vertebræ, mainly on the left side.

Rheumatic pericarditis is always a serious condition, although not often immediately fatal. The cases in which death occurs during the acute stage are almost invariably examples of a general carditis, that is to say, in addition to the pericardial inflammation, there is also an acute endocarditis, shown by the presence of a row of small recent granulations close to the free edge of the mitral valve and sometimes on the aortic cusps also, together with a diffuse myocardial inflammation demonstrable by microscopic examination. More commonly than not in children, there is no appreciable synovial inflammation. In cases which do badly there is rapidly increasing anæmia, the pulse is very frequent, and a crop of rheumatic nodules often develops; persistent vomiting is of most unfavourable augury, but the temperature is of little value in prognosis. Sometimes the downward course is extremely rapid, and no treatment seems to have the slightest effect in arresting the progress of the disease. Such a case has come under my notice since this article was commenced; the patient was a girl aged 11 years, she was only under treatment for ten days, during which period the temperature never rose above the normal and not the slightest pain was complained of, although loud friction was heard over the præcordial area. At the post-mortem examination there was very little free fluid in the pericardial sac, which was becoming obliterated by adhesions; there were small recent vegetations on the aortic, mitral and tricuspid valves.

Whilst the immediate danger to life of rheumatic pericarditis is by no means negligible, a still greater risk, and one from which there is practically no escape, is involved by the after-effects of the pericarditis upon the heart, owing to the adhesions to which it gives rise. It is still somewhat uncertain whether an attack of pericarditis can occur without setting up any adhesions; in rheumatic pericarditis this must certainly be very rare, and in the sub-acute attacks already referred to the tendency is for the disease to recur until the pericardial sac is completely obliterated, the pericardium itself becoming greatly thickened and eventually the seat of dense fibrous changes. Such changes must necessarily seriously hamper the heart's action, especially as inflammation and subsequent fibrosis are very likely to extend from the pericardium into the superficial layers of the myocardium. Nor is this all; rheumatic pericarditis rarely occurs as an isolated lesion in the heart, particularly in children; in the majority of cases mitral disease is either already present or an acute endocarditis develops simultaneously with the pericarditis, perhaps on the top of an old valvular lesion. Anyhow, the final result is mitral disease complicated by an adherent pericardium, a peculiarly unfavourable combination. In many, perhaps in the majority, of fatal cases of heart disease in childhood, the commencement of the downward course dates from an attack of pericarditis. Certainly the prognosis of any case of valvular disease is infinitely worse if the pericardial sac is also obliterated. It is, therefore, important to be able, if possible, to diagnose the condition, even though it may not materially affect our treatment. Diagnosis, however, is often by no means easy; the most important signs of adherent pericardium enumerated in books are—systolic recession at and around the apex-beat (recession limited to the apex-beat has no special diagnostic value when the heart is enlarged), systolic retraction of the chest wall behind, between the eleventh and twelfth ribs, absence of alteration by change of posture or by deep inspiration in the position of the apex-beat, or in the area of cardiac dullness. None of these signs, however, is absolutely characteristic or reliable, and probably they are only present, when, in addition to obliteration of the pericardial sac itself, there are external adhesions between the outer layer of the pericardium and the chest wall, a result of mediastino-pericarditis. Although the latter condition is no doubt the more serious of the two, it is desirable to be able, if possible, to diagnose the internal adhesions when present alone, although in the absence of a definite history of pericarditis we can rarely do so with absolute certainty. All we can say, perhaps, is this, that when we meet with a child with a greatly dilated heart, especially if large enough to give rise to well-marked præcordial bulging, and the physical signs point only to the existence of mitral disease, without any aortic lesion, under such circumstances the pericardial sac is probably obliterated, even though all the above-mentioned signs of the condition are absent. Another possible indication of adherent pericardium is the absence, without obvious cause, of improvement under treatment in a heart which is clearly failing. Certainly in a case of chronic valvular disease in a child or young adult, infective endocarditis being excluded, rest and cardiac tonics should effect at least a well-marked temporary improvement. Their failure to do so is found not infrequently on the post-mortem table to be attributable to an adherent pericardium.

Lastly, the question naturally arises whether, apart from those preventive measures which apply to all the various manifestations of rheumatism, anything can be done when once pericarditis has developed to diminish its severity and the likelihood of the formation of adhesions, with all their possibly disastrous consequences. It must be confessed that our powers in this direction are very limited. The remarkable action of drugs of the salicylate group upon rheumatic inflammation of joints is one of the most striking of therapeutic triumphs: how disappointing, therefore, it is to find that these same drugs have apparently very little influence upon rheumatic inflammation of the endocardium and pericardium; in fact fresh heart mischief sometimes actually arises while a patient is still remaining absolutely in bed and continuing to take salicylate of soda or an allied drug. Perhaps this is due to the impossibility of completely resting the heart as we can an inflamed joint. Nevertheless it seems only right to give one or other of these remedies, since we at present know of no other which offers the slightest hope of exercising any specific influence over a rheumatic inflammation, and if they do but little good neither are they likely to be harmful. The idea that, when pure, they are cardiac depressants is, I am convinced, quite erroneous; it is the rheumatism which is the depressant, not the drug; aceto-salicylic acid, in fact, seems to have a distinct tendency to slow and strengthen the heart. None the less this idea of a depressing action doubtless often leads to their being given in far too small doses, and so to a failure to obtain the benefits which might accrue from a bolder line of treatment. Cardiac tonics and stimulants, especially strychnine hypodermically, should of course be given as may seem necessary, but the value of digitalis to a heart the myocardium of which is inflamed is somewhat doubtful; it is often tried, but probably with little benefit. Views as to local treatment vary widely, but I believe the continuous application of an ice-bag to the præcordium, as advocated by Dr. Lees, is often useful, especially when pain is severe, and is more efficacious than the hot applications commonly employed. The value of blisters is exceedingly difficult to estimate, but probably they do good, and certainly the fact that they interfere to some extent with physical examination of the heart is not a sufficient reason for refusing to use them. Paracentesis pericardii is, as already mentioned, rarely required; moreover, there is no small risk of puncturing the heart when performing it.

In every case by far the most important treatment is complete and prolonged rest, in order to give the damaged heart the best possible chance of recovering itself before any unnecessary strain is placed upon it. That we are not able to rest the heart completely, as we can a joint, is certainly no argument for not trying to rest it at all, but rather one for giving it a still longer period of incomplete rest. Three months of absolute rest in bed or on a couch, with a course of massage and perhaps resistance movements towards the end of the period, when every indication of active mischief has subsided, and then another three months of partial rest, during which all active and prolonged exercise is forbidden, is certainly not too long a period after an attack of acute pericarditis, especially when we remember that the myocardium and the mitral valve have probably been damaged also. The after-consequences of the pericardial adhesions upon the heart must be treated on ordinary lines, that is, by the usual measures adopted for imperfect or failing cardiac compensation. The surgical intervention which has been suggested for the separation of adhesions between the pericardium and chest wall cannot be regarded at present as feasible and obviously can never be available for internal adhesions.

